# 

**Published:** 1898-04-23

**Authors:** 


					I THE HOSPITAL. "The standard of highest purity."?Lancet. Apeil 23rd, 1898.
CADBURY'S vjr ^ CADBURYS
1 " COCOA Inl mr* COCOA
cocoa ^1 ipif m; cocoa
ABSOLUTELY PURE. gk, l*jrt 1 NO DRUGS OR ALKALIES USED.
Lkahb Norwich hospital
No. 604. Vol. XXIV. mgSSiS Saturday,
April 23rd, 1898.
[Subsection Terns,] pBICE TWOPENCE.

				

